# Orbital metastases as first sign of metastatic spread in breast cancer: Case report and review of the literature

**DOI:** 10.1186/1758-3284-3-37

**Published:** 2011-08-22

**Authors:** André M Eckardt, Majeed Rana, Harald Essig, Nils-Claudius Gellrich

**Affiliations:** 1 Department of Cranio-Maxillofacial Surgery, Hannover Medical School, Carl-Neuberg-Strasse 1, 30625 Hannover/Germany

**Keywords:** Orbital metastasis, breast cancer, navigation-assisted surgery

## Abstract

**Background:**

Intraorbital metastases of breast cancer is rare with only 3-10% of all ocular metastases. We report a case of orbital metastases as first sign of systemic metastatic spread in a female patient with breast cancer.

**Methods:**

The patient had been diagnosed with breast cancer 3 years before. Her present complain was local pain, diplopia and periorbital swelling. A CT scan revealed extensive bony destruction of the orbital roof/anterior skull base. Bone scintigraphy demonstrated additional uptake at the level of the skull base, cervical spine, ilium and ribs suggesting metastatic spread to the skeleton. A navigation-assisted intraorbital biopsy from the orbital roof revealed a metastasis of breast cancer. With the confirmed diagnosis of metastatic breast cancer the patient was refered to the oncologist for further tumor staging. As further treatment she received systemic palliative chemotherapy in addition to intravenous treatment with bisphosphonates.

**Conclusion:**

In patients with a previous history of breast cancer who complain even of mild ophthalmologic symptoms such as local pain, periorbital edema, it is important to consider ocular or orbital metastatic disease. Adequate 3D-Imaging followed by a biopsy will usually confirm the diagnosis.

## Introduction

Orbital metastases occur in 2% to 3% of patients with cancer. Metastases of breast cancer account for the majority of ocular and orbital metastases [1]. Clinical signs and symptoms may include proptosis, diplopia, pain, exopthalmus, blurred vision and a visible or palpable tumor mass in the orbital or periorbital region. The overall prognosis of patients with orbital metastasis is generally poor. In 12-31% of the affected patients [2-4] eye metastases are the first sign of malignant disease or metastatic spread. Once the diagnosis has been established treatment is mainly with palliative intention and focuses on symptom relief and improvement of orbital function as long as possible.

We report a case in which orbital metastasis of breast cancer was diagnosed with a navigation-assisted surgical biopsy. Further clinical staging revealed previously unknown multifocal skeletal metastasis.

In addition diagnosis and treatment concepts of orbital metastases will be reported.

## Case report

A 73-year old woman presented with a 3-week history of left orbital pain with slight exophthalmus. Three years before the patient had been diagnosed with breast carcinoma cT4 cN1. The tumor was ER/PR positive and HER2-neu negative. The initial treatment consisted of neoadjuvant chemotherapy with epirubicin/cyclophosphamide (EC-protocol) followed by surgical tumor ablation and dissection of the left axilla. Postoperative external beam radiation of the thoracic wall was performed. After completion of the initial treatment with complete remission the patients further clinical course was uneventful. Due to the positive receptor status antihormonal therapy with anastrozole was initiated.

**Figure 1:**
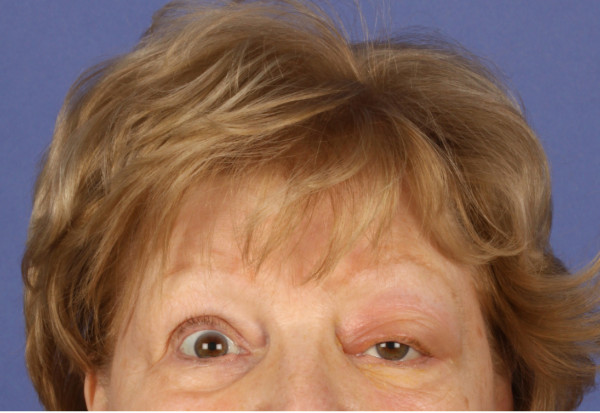
**73-year old patient with left orbital swelling and slight ptosis and proptosis**. Clinically an firm mass was palpable that persisted for about 3 weeks causing pain and diplopia.

**Figure 2:**
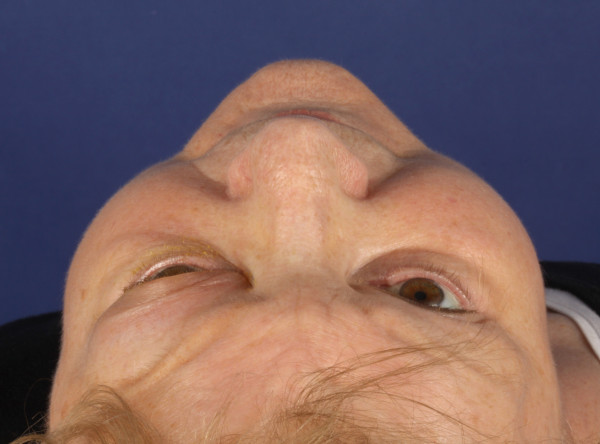
**Axial view shows left orbital swelling with ptosis and proptosis**.

**Figure 3:**
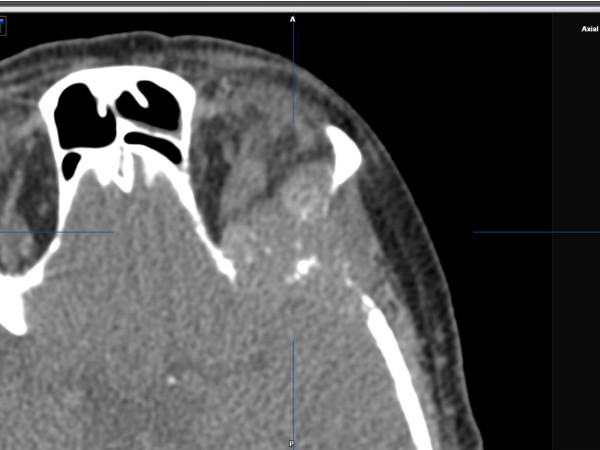
**The axial CT scan shows an infiltrating tumor mass with bony destruction of lateral orbital wall and skullbase**.

**Figure 4:**
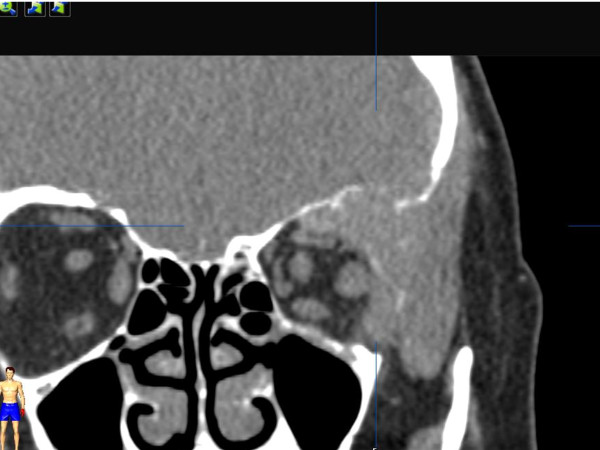
**The coronal CT scan reveals large tumor mass of the left orbit with bony destruction of orbital roof and lateral orbital wall as well as skullbase infiltration**.

**Figure 5:**
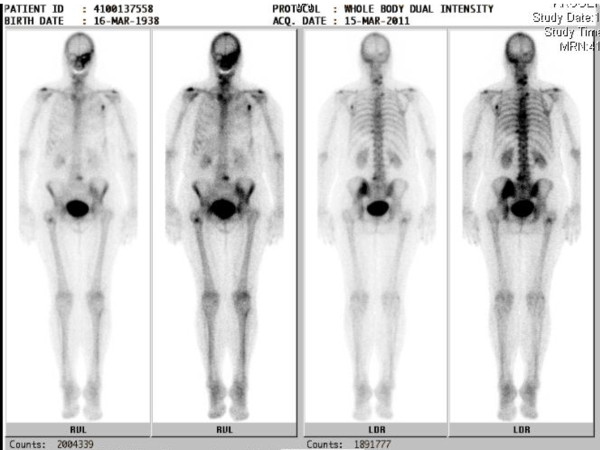
**Technetium-Bone scintigraphy reveals multiple osseous lesions in the skeleton suspicious for metastases of the breast cancer**.

**Figure 6:**
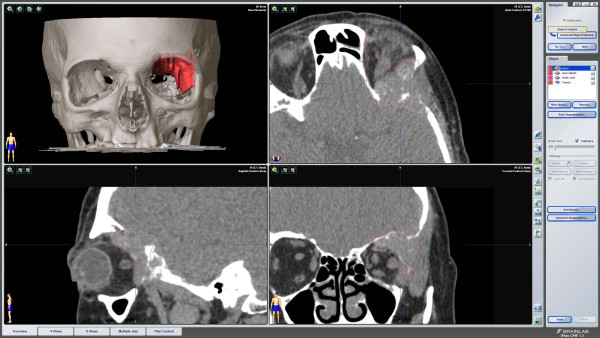
**Preoperative multiplanar imaging using the iPlan3.0 software (Brainlab^®^) allows for segmentation of the tumor area and virtual planning of the biopsy**.

**Figure 7:**
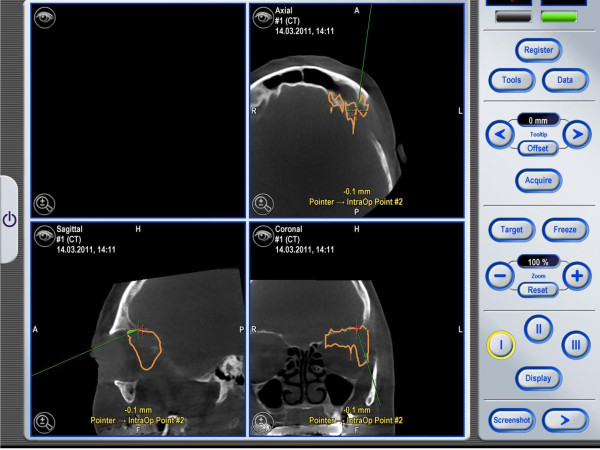
**The current position of the pointer tip is visualized by a green line in all reconstruction views in real-time**. The actual landmark or biopsy position is marked by a red cross. These positions are saved and could be transferred to planning data post-operatively.

In the clinical presentation we found left supraorbital swelling and exophthalmus. (Figure 1 and 2). The patient complained of left orbital pain and diplopia. There were no clinical signs of neuromuscular dysfunction of the eye or any other neurological disorder. CT imaging studies were suspicious for left orbital metastasis with a suprabulbar tumor mass, sized 3 × 4 cm, with osseous infiltration of the orbital roof and lateral orbital wall, frontal skull base and sphenoid wing (Figure 3 and 4). General restaging, which was then performed, showed multifocal osseous metastases of the skull base, cervical spine, right femur, left ilium and ribs (Figure 5). For diagnostic confirmation a biopsy taken from the left orbital roof was planned. As a first step for technical and surgical planning DICOM data of the patient were imported into the planning application iPlan3.0 (BrainLab^®^). (Figure 6). An MRI dataset was used to determine the position and shape of the tumor structure, while a CT dataset was used to determine the patients anatomical structure and to plan reference points for the surgery. The tumor shape itself is outlined manually by several tools for brushing. If its shape has been finalized, target points for taking a biopsy could be planned as well (Figure 7). Using an upper eyelid crease incision the tumor area was approached by blunt dissection. Intraoperative using pointer-assisted navigation the tumor region was approached and a representative snap biopsy was taken. The pathology report confirmed the diagnosis of a metastatic poorly differentiated invasive ductal carcinoma of the breast; further immunohistochemical staining demonstrated ER/PR positivity, positive staining for HER-2/neu and Ki67 Index of 40%. The postoperative course was completely uneventful with no ocular morbidity. The patient was discharged on the 3^rd ^postoperative day. She was referred to the oncologist for further systemic chemotherapy and bisphosphonate treatment. Currently she receives hormonal therapy with daily tamoxifen 20 mg; in addition bisphosphonate treatment with 4 mg zolendronate/monthly was initiated. Local radiotherapy of the left orbit and skull base will be reserved in case of progression of ocular symptoms.

## Discussion

Typical manifestations of orbital metastases include a palpable mass causing displacement or proptosis of the globe, pain, inflammation, bone involvement, chemosis, and eyelid swelling [5].

Metastases to the eye and orbit have been described for a variety of solid tumors, but breast carcinoma accounts for the majority of ocular and orbital metastases [6-9]. In breast cancer - the most frequent cause of cancer-related death in women [10] - orbital metastases have been described in large studies in the range from 28.5-58.8% [1,11,12]. Orbital metastases from breast cancer tend to infiltrate the extraocular muscle and surrounding orbital fat, causing motility deficits. The histology features of adenocarcinoma of the breast vary, and the histologic features of orbital metastases may differ from the primary tumor.

Patients who have been diagnosed with orbital metastases usually carry a poor prognosis: the mean survival after diagnosis of such metastases is 31 months (range, 1-116 months) [13]. Even if the orbit is the only clinical suspected site of metastatic involvement, the likelihood of further distant metastases in other organs is high as was shown in our patient following further staging procedures.

Treatment of orbital metastases aims at improving patient's quality of life and restore or preserve visual function. Usually treatment of orbital and ocular metastases is palliative and may include radiotherapy, systemic chemotherapy, hormonal therapy, or surgery in selected patients.

Radiotherapy is the mainstay for orbital and ocular metastases and appears to be safe and effective with objective response rates up to 79% [14]. Radiotherapy improves symptoms in 80% of cases and restores vision in some cases [15]. However, cataract formation and radiation retinopathy are potential side effects of external beam radiotherapy; these potential sequelae must be balanced against the overall prognosis and survival of the individual patient [12,16]. Systemic chemotherapy is another mainstay in the palliative setting of orbital metastases; in addition the initiation of bisphosphonate treatment is important if osseous metastasis have been diagnosed. Novel targeted systemic treatments are increasingly used in addition to cytostatic chemotherapy. Recently, one case of dramatic local response of a unilateral choroidal metastasis in Her2/neu-positive breast cancer to systemic therapy with trastuzumab and vinorelbine was reported [17].

In general, extensive orbital surgery to remove the metastasis is not recommended as this is not curative and may be associated with significant ocular morbidity [15]. Enucleation or rather radical measures offer no advantages in terms of progression or survival and should only be used in cases of intractable ocular pain or unmanageable local hygiene due to rapid tumor growth [5]. The only appropriate surgical intervention for breast carcinoma metastatic to the orbit is a biopsy to establish the diagnosis [5].

In summary, although rare, breast cancer patients can develop metastases to the orbital and ocular region. Patients with a history of breast cancer presenting with ocular symptoms such as ptosis, proptosis, diplopia, pain, exophthalmus should be evaluated for orbital metastases. Once diagnosis is confirmed treatment for patients with orbital metastases is multidisciplinary.

## Competing interests

The authors declare that they have no competing interests.

## Authors' contributions

AME made substantial contributions to conception and design of the manuscript as well as data acquisition. MA, HE have been involved in drafting the manuscript. NCG was involved in revising the manuscript. All authors read and approved the final manuscript.

## Consent statement

Written informed consent was obtained from the patient for publication of this case report and accompanying images. A copy of the written consent is available for review by the Editor-in-Chief of this journal.
